# Potential influence of long-term medication on physical performance in the context of long-term rehabilitation process in soldiers: a retrospective cohort analysis

**DOI:** 10.3389/fresc.2025.1605767

**Published:** 2025-11-18

**Authors:** Jennifer-Daniele Schmitz, Roman Korte, Andreas Lison, Joachim Gerß, Christoph Schulze

**Affiliations:** 1 Orthopaedic Clinic and Polyclinic, Rostock University Medical Center, University of Rostock, Rostock, Germany; 2 The Bundeswehr Center of Sports Medicine, Warendorf, Germany; 3 Institute for Biostatistics and Clinical Research, University of Münster, Münster, Germany; 4 Institute of Physical Medicine and Rehabilitation, Paracelsus Medical University, Salzburg, Austria

**Keywords:** rehabilitation, physical performance, bicycle ergometry, long-term medication, polypharmacy, drug-drug-interaction

## Abstract

**Objective:**

Military service is demanding and requires maintenance of optimal physical and mental health. Deployment-related incidents can precipitate the onset of physical and/or psychological trauma necessitating appropriate pharmacological intervention. However, current strategies for medical vocational rehabilitation do not include systematic medication assessment.

**Methods:**

This study analyzed physical performance and long-term medication use in middle-aged soldiers diagnosed with physical impairments and/or posttraumatic stress disorder. Patient records were analyzed to investigate the number of drugs, polypharmacy, drug–drug interactions and drugs targeting the nervous system in relation to bicycle ergometry performance.

**Results:**

A total of 172 patients were inclusion, with 45 patients (26.6%) receiving comprehensive medication. Patients with posttraumatic stress disorder, who were taking an average of 3.0 medications each, demonstrated significantly lower maximum performance than did the controls [*n* = 93, 217 (200–250) W vs. *n* = 34, 250 (225–267) W, *P* = 0.006]. Polypharmacy [*n* = 42, 217 (200–250) W vs. *n* = 34, 250 (225–267) W, *P* = 0.017] and drug–drug interactions [*n* = 49, 221 (200–250) W vs. *n* = 34, *n* = 34, 250 (225–267) W, *P* = 0.024] negatively impacted maximum performance, with patients experiencing 1.6 interactions on average of varying severity. A significant negative correlation was observed between the number of drugs, anatomical-therapeutic-chemical classification, polypharmacy, number, and severity of drug–drug interactions and poor maximum performance. Although no other significant differences were noted across all groups, posttraumatic stress disorder patients without medication achieved significantly greater performance than did somatic patients [*n* = 34, 250 (225–267) vs. *n* = 33, 217 (200–250), *P* = 0.009].

**Conclusion:**

In conclusion, highly prevalent polypharmacy may contribute to physical performance limitations, highlighting the need for further research and professional support in medication management within rehabilitation programs for mental illnesses.

## 1 Introduction

Medication management has shown that highly complex medication in the geriatric population is associated with an increased risk of drug-related problems (DRPs) (1, 2). A DRP encompasses a spectrum of events or circumstances that either actually or potentially impede desired health outcomes within drug therapy (3). Over time, various scenarios of DRP patterns have been developed, including but not limited to “unnecessary drug use” and “medication use without indication”. Polypharmacy, associated with triggering DRP, denotes concurrent long-term use of two or more drugs (4). It is linked to the risk of adverse outcomes such as falls, frailty, disability, and mortality in older adults. Consequently, the optimization of medication regimens is one of the pivotal components of comprehensive geriatric care (4). However, little is known about DRPs and their effects on desired health outcomes in middle-aged patients with comorbidities (5). The guideline set forth by the German Society of General Practice and Family Medicine (DEGAM) delineates several factors contributing to polypharmacy (6). These include co- and multimorbidity requiring multidrug therapy, inadequate communication between healthcare providers (inter- and intraprofessionally) and noncalculated self-medication, which limits patient adherence to prescribed regimens (6). Symptoms arising from polypharmacy are predominantly nonspecific upon identification and may ultimately result in functional decline in later stages (6). Studies conducted in the geriatric population have shown that polypharmacy serves as a potential precursor to functional impairment, manifesting as reduced physical functionality and compromised performance (7). In turn, there is ongoing discourse regarding targeted interventions aimed at increasing physical performance within this patient demographic as an integral component of an expanded repertoire range of somatically oriented rehabilitation strategies (8, 9). Mental comorbidities are defined as the co-occurrence of a mental disorder alongside a physical ailment and commonly manifest in the presence of somatic diagnoses pertinent to rehabilitation (10, 11). Notably, a recent study reported that approximately one-fifth of patients who underwent somatic rehabilitation concurrently presented with a mental disorder (11). Military-based health research has reported that approximately 2%–3% of returning soldiers are diagnosed with symptoms of trauma post deployment (12). Furthermore, it has been postulated that experiences related to deployment, particularly those stemming from intense combat operations, are linked to a significantly greater prevalence of mental disorders (13, 14). In the context of medical vocational rehabilitation (MVR), mental comorbidity holds substantial relevance due to its potential to adversely impact motivation and active engagement in the rehabilitation process (10). Therefore, the diagnosis and treatment of mental comorbidities have garnered increased attention in rehabilitation science (8, 9, 15). Individually defined therapeutic goals have been pursued through established medical and occupational rehabilitation measures within an interdisciplinary team (16, 17). The components of MVRs include requirement-oriented diagnostics, intensified social and vocational counseling, participation in work-related psychosocial groups and workplace training (16). Although systematic medication reviews are currently not integrated into these components, guidelines on mental comorbidity issued by the German Pension Insurance address this aspect (18). This guideline recommended documenting current medication prescribed during psychotherapeutic treatment, focusing on type, extent and efficacy. Models elucidating mental comorbidities in the context of underlying somatic diseases have identified medication as a possible risk factor for mental disorders (10). Following physical and/or psychological trauma, medication plays a supportive role in therapy at various angles. Although the use of antidepressants and antipsychotic medications may influence military readiness for duty, medications affecting other organ systems, such as the endocrine, cardiovascular or vascular systems, carry potential risks related to adverse drug reactions (ADRs) and physical performance (7, 19). Performance diagnostic parameters play a crucial role in objectively assessing the prognosis of rehabilitation (20). This is particularly essential in the context of the military, where physical performance is a professional requirement.

The aim of this study was to retrospectively record the prevalence of long-term medication (LTM) usage among physically and/or mentally injured military personnel at the onset of MVR. Our aim was to investigate potential drug–drug interactions (DDIs), particularly in the context of polypharmacy, and to ascertain any correlation between medication profiles and performance outcomes.

## 2 Methods

### 2.1 Ethics approval

Ethical approval for conducting the study was granted from the Ethics Committee of the Medical Faculty of the University of Rostock as well as the Ethics Committee of the Westphalia-Lippe Medical Association and the Westphalian Wilhelms University of Münster (Ethics ID 2020-497-b-S, approval 25th July 2020). The study was carried out in accordance with the institutional research committee's ethical standards.

### 2.2 Study design

This retrospective cohort study enrolled active and former soldiers of the German Armed Forces who were diagnosed with a primary complex impairment of the musculoskeletal system following wounding, injuries or severe orthopedic illnesses and/or a primary psychological disorder indicative of posttraumatic stress disorder (PTSD). Patients underwent an initial assessment followed by a three-week MVR. Patients were categorized into an intervention group receiving LTM and a control group not receiving medication based on their medication status. Subgroups were further delineated based on the primary diagnosis. Regardless of their medication status, all patients included exhibited at least one physical impairment. Medication considered for inclusion was prescribed for preventing or alleviating a chronic illness persisting for at least 3 months, while medication used for a case of need that had not yet occurred or taken without continuity was not considered. Data collection spanned from 2011 to 2020, with assessments carried out at the initiation of rehabilitation, encompassing performance diagnostic evaluation conducted subsequent to initial interviews. The inclusion criterion included soldiers demonstrating a significant risk to fitness for duty or ability to perform previous occupational tasks. The exclusion criteria included patients lacking a requirement for somatic rehabilitative measures, those diagnosed with cancer or mental illnesses other than PTSD, and those lacking performance diagnostic assessments.

### 2.3 Survey sample

A total of 381 patient records were analyzed. In this study, the use of at least three long-term medications was defined as the cutoff for polypharmacy (4). Medication data included the name, dosage and frequency of administration of the active ingredient. Drug-induced interactions were identified using Scholz database (as of 2021/22) and were then categorized as moderate or severe by applying the proprietary traffic light system of the software. Additionally, interactions were classified based on the international consensus of potential clinically significant drug interactions in the elderly population (21). To distinguish between the effects of drugs with differing primary mechanisms, medications were categorized into their respective anatomical groups utilizing ATC. Notably, drugs categorized under ATC group N were selected for comparison against other groups. Somatically focused rehabilitation interventions included individually tailored sports programs for all patients based on performance diagnostic examinations.

### 2.4 Cycling ergometry

The maximum performance per kilogram body weight [p-max. in Watts (W)] and performance at lactate level of 4 mmol/L in capillary blood [p-4-mmol/l-Lac. (W)] were assessed through bicycle ergometry. The bicycle ergometer “Schiller ERG 911 S plus” (Schiller Medizintechnik, Feldkirchen, Germany) was used with load adjustments following the German Association for Sports Medicine and Prevention guidelines, employing a step profile with a 50-Watt increase every three minutes (22). Maximum performance was defined as the point of test termination based on subjective stress limits or other predefined termination criteria. The capillary blood lactate concentration was monitored at three-minute intervals throughout the test (21) with a detailed procedure previously described elsewhere (21). Using the “Winlactat” software (Mesics GmbH, Münster, Germany), both the maximum power output and the power at which the patient surpassed the threshold of 4 mmol/L lactate (p-4-mmol/l-Lac.) were calculated through regression analysis.

### 2.5 Statistical analysis

The data were checked for normal distribution through Q–Q plots and Shapiro–Wilk tests. Descriptive and inferential nonparametric procedures were utilized for statistical analysis. In the descriptive analysis, sample size (N), standard deviation (SD), median (Mdn) and the interquartile range (IQR) of the spiroergometric measurement data were assessed relative to medication at the time of initial rehabilitation with documented performance data thereafter. Differences in performance within and between medication groups were examined using Mann–Whitney *U* and Kruskal–Wallis tests with multiplicity adjustment according to Bonferroni correction. The associations between diagnostic performance and medication use were investigated using Spearman bivariate correlation analyses. The correlation strength was interpreted according to Cohen (22). The local significance level was set to α = 5%. Two-sided *P*-values were considered to be noticeable (“significant”) in case *p* ≤ 0.05. The software SPSS Statistics Version 27 (IBM, Armonk, USA) and Scholz database (ePrax GmbH, Munich, Germany) were used to perform statistical analyses, whereas GraphPad Prism 6 was used to create the figures.

## 3 Results

Out of the initial pool of 381 patients, 172 (males: 164/females: 8) met the specified inclusion criteria. A total of 106 (61.3%) patients had fully documented medication, whereas the control group consisted of 66 (38.7%) patients without medication. A comprehensive medication regimen (polypharmacy) was noted for 45 patients (26.6%). The mean age of the patients was 36 ± 8 years at the time of recruitment. On average, patients weighed 93.5 ± 17.4 kg and stood at a height of 180 ± 7 cm, resulting in an average body mass index (BMI) of 28.8 ± 4.6 kg/m^2^. The average waist-to-height ratio (WHtR) was 0.54 ± 0.07. Regarding bicycle ergometry, the average p-max was 229 ± 48 W, while the p-4 mmol lactate was 162 ± 39 W. While detailed characteristics of the subgroups and corresponding comparisons are presented in Table 1, it should be noted that 11 of 127 patients (8.7%) in the PTSD group and 6 of 45 patients (13.3%) in the somatic trauma subgroup were additionally diagnosed with comorbid depression.

**Table 1:** Characteristics of patients enrolled (*n* = 172).

Variables	PTSD (*n* = 127)	Somatic trauma (*n* = 45)	*P* value
Age (years)	37 (33–43)	29 (25–37)	<.001***
Weight (kg)	95.7 (87–104.5)	88.2 (79.6–96.4)	.002**
Height (cm)	180 (176–185)	178.(173–185)	.273
BMI (kg/m^2^)	29.4 (26.6–32.0)	26.9 (25.1–29.80)	.001***
WHtR	0.56 (0.52–0.62)	0.52 (0.50–0.57)	.010**
p max [W]	225 (200–258)	217 (200–215)	.116
p-4 mmol Lac. [W]	161 (138–184)	151 (127–174)	.091
Gender			
Male (N)	121 (69.9%)	43 (24.9%)	
Female (N)	6 (3.5%)	2 (1.2%)	
Long-term medication			
Yes (N)	93 (53.8%)	13 (7.5%)	
No (N)	34 (19.6%)	33 (19.1%)	
Polypharmacy			
Yes (N)	42 (24.3%)	3 (1.7%)	
No (N)	51 (29.5%)	10 (5.8%)	

Data presented as median (Mdn), interquartile range (IQR), and number (N).

** Comparison is significant at 0.01 level (two-tailed).

*** Comparison is significant at the 0.001 level (two-tailed); *P* value: Mann–Whitney *U*-test.

### 3.1 Impact of long-term medication on performance in the study population

**Figure 1:**
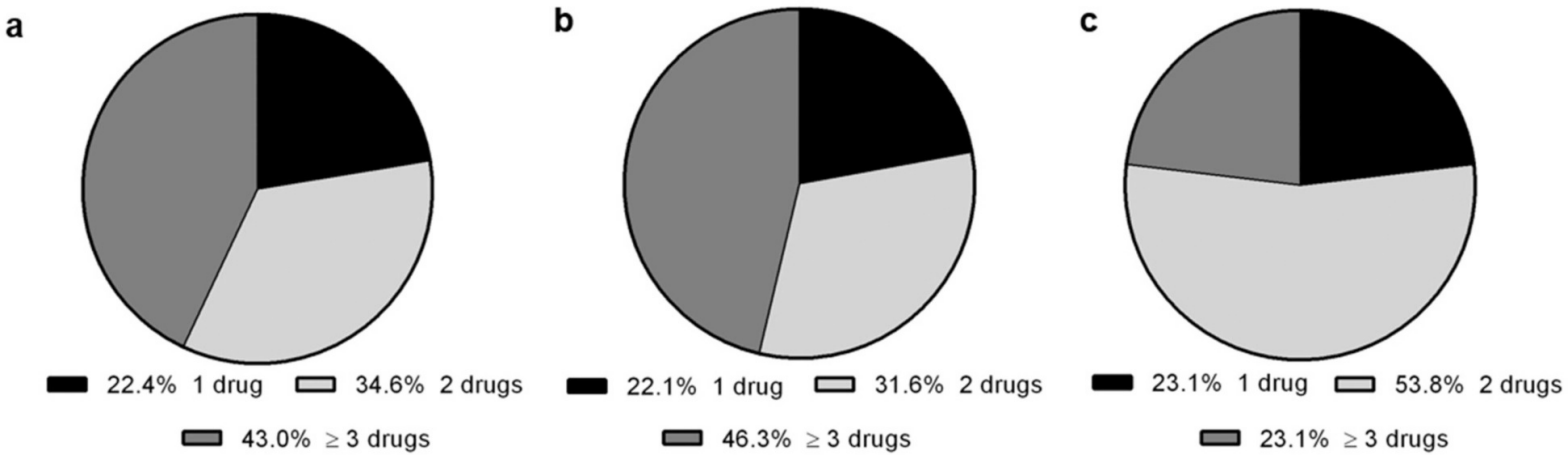
Number of drugs taken on a long-term basis as parts per whole (%). **(a)** Distribution in the overall study cohort (*N* = 314), **(b)** Distribution in the PTSD group (*n* = 283), **(c)** Somatic group (*n* = 31).

A total of 314 drugs were identified to be taken on a long-term basis, with an average of 2.9 (±2.0) drugs per patient. The drugs were distributed as shown in Figure 1a.

Upon analyzing performance data across the entire study population, no significant differences were detected in terms of the maximum power output (p-max) or maximum lactate threshold (p-4 mmol Lac.) concerning the use of LTM. However, among patients taking ≥ 3 drugs, a significant reduction in maximum performance was evident compared to those not taking any medication [*n* = 45, 217 (200–233) W vs. *n* = 67, 225 (208–267) W, *P* = 0.041]. Maximum performance correlated negatively with the number of long-term drugs with low strength (Table 2).

**Table 2:** Spearman correlation of p-max. and p-4-mmol Lac. and specific medication factors.

Variables	Spearman correlation statistics	1	2	3	4	5	6
Reha-Cohort							
p-max.	Spearman rho	−.168*	−.161*	−.123	−.129	−.107	−.147
	Sig. 2-tailed	.027	.034	.108	.117	.194	.073
	n	172	172	172	149	149	149
p-4-mmol Lac*.*	Spearman rho	−.055	−.067	−.041	−.044	.027	.106
	Sig. 2-tailed	.481	.388	.603	.603	.749	.210
	n	166	166	166	143	143	143
PTSD							
p-max.	Spearman rho	−.287**	−.281**	−.243**	−.237*	−.210*	−.238*
	Sig. 2-tailed	.001	.001	.006	.014	.031	.014
	n	127	127	127	106	106	106
p-4-mmol Lac.	Spearman rho	−.127	−.145	−.112	−.079	−.014	−.218*
	Sig. 2-tailed	.165	.112	.221	.434	.893	.029
	n	121	121	121	101	101	101
*Somatic trauma*							
p-max.	Spearman rho	−.056	−.056	−.003			
	Sig. 2-tailed	.713	.710	.982			
	n	46	46	46			
p-4-mmol Lac.	Spearman rho	−.015	−.017	−.011			
	Sig. 2-tailed	.920	.911	.941			
	N	45	45	45			

1 = number of drugs taken; 2 = number of ATC groups to which drugs are assigned; 3 = polypharmacy; 4 = potential DDI; 5 = interaction intensity; 6 = number of DDIs.

* Correlation is significant at 0.05 level (two-tailed).

** correlation is significant at the 0.01 level (two-tailed).

### 3.2 Long-term drug use and interactions in the PTSD group

**Figure 2:**
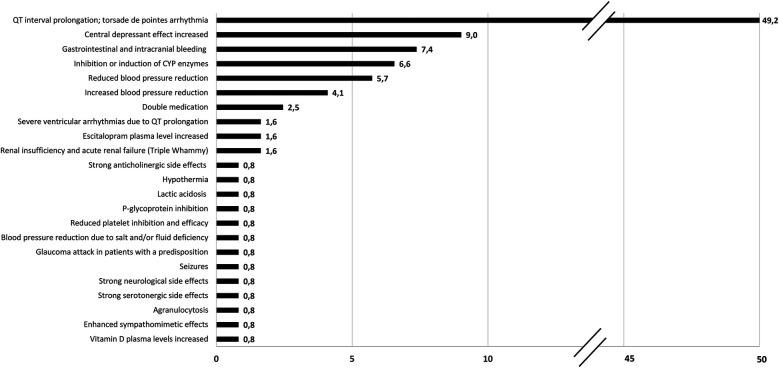
Effects due to potential DDIs and the frequency of their occurrence in the PTSD subgroup. The data are presented as percentages (%).

A total of 283 long-term drugs were reported in this group, averaging 3.0 (±2.0) medications per patient. The distribution of these drugs is depicted in Figure 1b. 72 patients were identified with medication comprising at least two long-term drugs, resulting in 122 potential drug interactions. Of these interactions, 81 were classified as potentially moderate, and 41 were classified as potentially severe. On average, each PTSD patient experienced 1.6 (±1.4) drug interactions. 49 patients (68.1%) were at high risk of experiencing an interaction due to their medication, whereas 31.9% of the drug combinations posed a low risk. Notably, 76.6% of the entire PTSD group had at least one potentially moderate drug interaction, and 53.2% had at least one potentially severe drug interaction. Additionally, among the 72 patients receiving multiple medications, 17 (23.6%) exhibited potential interactions of both moderate and severe severity. Figure 2 illustrates the potential effects of DDIs and their frequency of occurrence in PTSD patients.

### 3.3 Performance in relation to medication in the PTSD group

**Figure 3:**
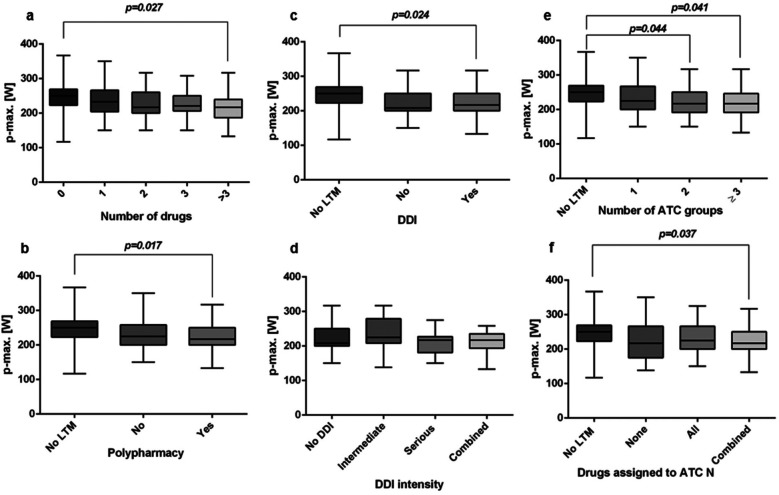
Boxplots illustrating the comparison of archived p-max values depending on the **number of drugs (a)** (*n* = 34, 250 (225–267) W vs. *n* = 21, 233 (208–266) W vs. *n* = 30, 217 (200–258) W vs. *n* = 14, 221 (200–250) W vs. *n* = 28, 217 (183–242) W, *P* = 0.027); **polypharmacy (b)** [*n* = 34, 250 (225–267) W vs. *n* = 51, 225 (200–258) W vs. *n* = 42, 217 (200–250) W, *P* = 0.017], **presence of DDI (c)** [*n* = 34, 250 (225–267) W vs. *n* = 23, 221 (200–250) W vs. *n* = 49, 221 (200–250) W, *P* = 0.024], **DDI intensity (d)** (*n* = 23, 221 (200–250) W vs. *n* = 17, 242 (208–275) W vs. *n* = 18, 211 (183–225) W vs. *n* = 14, 210 (200–233), *P* = 0.082), **number of ATC groups (e)** (*n* = 34, 250 (225–267) W vs. *n* = 43, 225 (200–267) vs. *n* = 25, 217 (200–250) W vs. *n* = 25, 210 (200–242) W, *P* = 0.041) and **drugs assigned to ATC N (f) (***n* = 34, 250 (225–267) W vs. *n* = 9, 217 (200–233) W vs. *n* = 37, 225 (200–266) W vs. *n* = 47, 217 (200–250) W, *P* = 0.037) in the PTSD subgroup. (LTM = long-term medication; the Kruskal–Wallis test for independent samples and Bonferroni correction were applied, and comparisons were significant at the *P* < 0.05 level.

PTSD patients on medication exhibited significantly poorer maximum performance unmedicated patients (Figure 3). The presence of medication was inversely correlated with maximum performance, albeit weakly (Table 2). Furthermore, analysis of LTM intake indicated that patients using more than three LTMs performed significantly worse than controls (Figure 3 and Table 2). This decline in maximum performance was observed to be proportional to an increase in the number of drugs taken.

Classifying active substances according to the ATC criteria revealed that combining substances from two different ATC groups significantly worsened the maximum performance compared to no medication. However, no additional significant differences in maximum power were observed when using active substances from three or more groups (Figure 3).

The occurrence of polypharmacy was indicated to be a potential influencing factor, with formulations containing three or more active ingredients showing a significant effect (Figure 3). The maximum performance correlated negatively with the presence of polypharmacy in a low manner (Table 2). Since polypharmacy is associated with an increased risk of DDI, potential DDIs were identified and categorized. Among individuals with PTSD, those exposed to a DDI demonstrated significantly inferior maximal performance compared to controls taking fewer medications. Moderate to severe interactions did not yield statistically significant disparities in maximal performance (Figure 3). No significant difference in performance was observed with p-4 mmol-Lac compared to other medication categories tested.

### 3.4 Performance in relation to medication in the group with somatic impairment

A total of 31 drugs, with an average intake of 2.4 (±1.4) drugs per patient, were identified among individuals with somatic diseases and LTM (Figure 1c).

There were no significant differences in p-max or p-4 mmol-Lac between the two groups in terms of maximum performance on bicycle ergometry. compared to the no-medication group (Table 3). Moreover, there were no statistically significant differences in p-max or p-4 mmol-Lac. those taking either 1–3 or >3 long-term medications were compared to those in the no-medication group.

**Table 3:** Comparison of cardiorespiratory performance parameters p-max and p-4-mmol-Lac. in patients assigned to the PTSD and somatic disease group.

Medication							
Yes				No			
p-max*.*							
	n	Mdn	IQR	n	Mdn	IQR	*P value*
PTSD	93	217	200–250	34	250	225–267	.006**
Somatic trauma	13	208	200–233	33	217	200–250	.722
* P value*		.601			.009**		
p-4 mmol-Lac-							
* *PTSD	88	160	133–178	33	171	148–191	.084
* *Somatic trauma	12	137	130–175	33	156	125–173	.909
* P value*		.356			.126		

Data presented as median (Mdn), interquartile range (IQR), and number (N).

** Comparison is significant at the 0.01 level (two-tailed); *P* value: Mann–Whitney *U*-test.

### 3.5 Comparison of performance values archived in PTSB and somatically impaired patients

As shown in Table 3, the comparison of the maximum performance achieved between PTSD patients and somatically impaired patients revealed no significant difference. In addition, maximum performance did not differ significantly in the presence of medication. Among patients without medication, those with PTSD achieved significantly greater maximum performance values compared to the somatic group at baseline.

The comparison of performance at a 4 mmol/L lactate concentration in capillary blood indicated no significant difference, which did not change depending on the LTM.

## 4 Discussion

### 4.1 Statement of key findings

We observed a notable decline in maximum performance among PTSD patients taking LTM. Notably, patients with greater medication complexity, such as those on multiple drugs or experiencing polypharmacy and DDIs, exhibited diminished performance levels. This suggests a perceived hindrance to rehabilitation efforts.

### 4.2 Interpretation

Recent findings in the literature highlighted significantly decreased physical fitness in depressive patients compared to healthy controls, which could not be explained by BMI, age, sex, or physical activity (23). However, our study revealed a distinct pattern among PTSD patients on LTM. Specifically, these individuals displayed markedly lower maximum performance levels and exhibited lower blood lactate concentrations than PTSD controls. Our findings indicate that factors such as age, weight, height, WHtR, and BMI did not significantly influence the observed decline in maximum performance. This result aligns with recently published literature in this area (23). These findings suggest that neither the presence of PTSD nor the anthropometric characteristics examined are primary determinants of reduced maximum performance.

As we focused on medication, we attributed the observed differences in performance primarily to a combination of medication-related factors, as illustrated in Figures 2, 3. These factors will be thoroughly examined and discussed.

### 4.3 Subgroup PTSD

#### 4.3.1 Number of drugs

Medication presence, regardless of severity, correlated negatively with maximum performance. The utilization of more than three long-term drugs was associated with a significantly poorer maximum cycling ergometric performance compared to medication-free PTSD patients. Furthermore, a negative correlation existed between the number of LTM and maximum performance, irrespective of the drug group. Consistent with previous research on interaction potential and medication management, it is widely acknowledged that the risk of interaction potential and the complexity of overall medication regimens, which are associated with adverse drug-related problems, increase with the number of long-term drugs prescribed (24, 25).

Notably, administering a single drug indicated for the treatment of psychiatric impairment did not affect performance. This suggests that antidepressive monotherapy does not adversely impact fitness restoration, which is a key rehabilitation goal.

While the hypothetical effects of medication on performance remain debated, there is an increased incidence for psychotropic drugs to be associated with increased cardiovascular complaints and reduced physical activity (26). Prescription cascades for the treatment of newly occurring complaints can lead to drug-related problems (27), which can lead to a loss of performance, among other effects.

Despite the well-known adverse effects of antidepressants and psychoanaleptics on performance, data supporting their beneficial effects are limited. However, selective serotonin reuptake inhibitors (SSRIs) have been reported to preserve physical ability (PA) (28), primarily based on pharmacological mechanisms and the dosage of the respective drug (25, 29).

The current German PTSD treatment guideline recommends three antidepressant drugs that target the serotonergic system (30). Among these, two are explicitly approved for PTSD treatment in Germany (29). However, these medications are associated with drawbacks, including delayed onset of action and limited efficacy in certain patients. Even dose escalation does not consistently yield the desired outcomes and may be accompanied by adverse effects (31).

In clinical practice, various antidepressants are commonly employed to alleviate PTSD symptoms (32), exerting different antidepressant effects and modulating psychomotor activity depending on their pharmacological profile (33). The diversity within the antidepressant drug class extends to their therapeutic effects and positive and negative side effects due to structural properties. Consequently, inappropriate combinations of these medications may compromise performance (34).

#### 4.3.2 Drug–drug interactions and polypharmacy

PTSD patients who were prescribed at least two LTM exhibited an average of 1.6 (±1.4) drug interactions per patient, as determined through the assessment of weighted interaction risks. Notably, this finding aligns with the results of a study on potentially clinically significant interactions in prescribed pharmacotherapy (35). However, demographic and medication differences between this cohort and the subjects referenced must be taken into account. With an average age of 66.3 years and an intake of six medications in the referenced cohort, the subjects were approximately twice as old, took twice as many medications, and experienced approximately twice as many DDIs compared to the subjects in this study. This observation implies that, in addition to the recognized risk associated with an increased number of medications, younger patients may also be equally susceptible to DDIs. However, it is important to note that the assumption of linearity, where a twofold increase in the number of medications leads to a twofold increase in DDIs, could not be validated by our study results. Therefore, predicting the extent of potential DDIs solely based on the number of medications remains uncertain.

Although our study could not conclusively determine the impact of DDIs on physical performance, it indicates the importance of identifying drugs that promote interactions. This is crucial for patients with altered physiology and a high medication burden, including younger individuals, to mitigate adverse effects. This finding is particularly significant for professions such as the military, where maintaining fitness and optimal medication use are paramount for duty and long-term military careers.

Studies within military environments are relatively underrepresented. In a study involving US veterans in nursing homes with depressive illness and antidepressant treatment, six out of ten residents encountered at least one prescription-related issue, with one in four experiencing at least one DDI (35).

Given the lengthy treatment durations for depressive and/or PTSD-related conditions, with pharmacotherapy being fundamental, it is crucial to pursue treatments with minimal side effects, especially for military personnel who require optimal physical and mental health.

The drug interactions quantified in this study, delineated according to their potential effects on physical performance and summarized by organ system (Figure 2), underscore the importance of medication review for middle-aged patients undergoing rehabilitation. This also prompts further investigation, as identifying and addressing drug interactions and unfavorable combinations is the first step toward structured medication optimization.

Dosage interval of drugs targeting the central nervous system:

Drugs targeting the nervous system (ATC N) that are administered late in the evening or at night can induce central depressant effects, leading to dose-dependent adverse reactions such as daytime drowsiness (36). Additionally, they elicit anticholinergic effects characterized by difficulties in concentration, dry mouth, light sensitivity, or orthostatic dysfunction, causing substantial discomfort (37). The antidepressant effect and risk of severe adverse effects, such as cardiac arrhythmias or anticholinergic symptoms, correlate with serum levels of active ingredients (38, 39). This was observed with tricyclic antidepressants such as amitriptyline, which patients in this study were prescribed. Amitriptyline is approved for major depression treatment in adults and for treating neuropathic pain, with recommended dose ranges varying depending on the indication (40).

The coadministration of a psychotropic drug with a drug from another ATC category in PTSD patients is associated with a negative effect on cycling ergometric performance (Figure 3f). It is plausible that concurrently administered active substances exert their effects via a common receptor (35). Consequently, pharmacodynamic interactions, which may manifest competitively, synergistically, or antagonistically, were possibly anticipated (35). Moreover, pharmacokinetic interactions occurring during drug transit through the body to their respective sites of action can alter drug plasma concentrations and precipitate ADRs (41).

### 4.4 Subgroup somatic impairment

In addition to the observed findings in PTSD patients, there was no significant decrease in maximum performance among patients with somatic impairments (Table 3), suggesting no medication-induced effects on performance. While pain can discourage physical activity and pharmacological interventions may improve mobility to some extent, weakness and fatigue present obstacles to mobilization (41, 42).

Consequently, the impact of LTM on the physical performance of our patients may neither be detrimental nor advantageous and may vary depending on impairment severity and musculoskeletal impairment.

### 4.5 Comparison of PTSD patients and somatic groups

The study showed that PTSD patients achieved significantly greater maximum performance values than patients in the somatic group did compared to primarily somatically impaired patients without existing LTM (Table 3), but there was no significant difference at the 4 mmol/L lactate threshold. Anthropometric data comparison revealed that somatically impaired patients were significantly younger and lighter than PTSD patients were (Table 1). This is plausible considering that orthopedic complaints are associated with physical impairment, leading to restricted p-max in somatically impaired patients, while PTSD patients do not exhibit primary physical limitations.

### 4.6 Strengths and limitations

To the best of our knowledge, this is the first retrospective cohort study to identify LTM and assess the potential relationship between medication and physical performance in middle-aged patients undergoing rehabilitation. Nevertheless, the present study has limitations.

First, data were retrospectively extracted from paper-based patient files with medication examined in terms of existing LTM, number of medications and the presence of polypharmacy and drug interactions. As there was no patient contact at any time during the data collection, it was not possible to carry out a comprehensive medication review. However, the factors mentioned are possible influencing factors that are strongly associated with a negative effect on the overall performance outcome (24, 33, 35, 43, 44). Future studies should include patient interviews that systematically examine influencing factors to include and further assess any deviations.

Secondly, the German armed forces' healthcare system involves multiple interfaces, potentially leading to communication challenges and diverse drug-related issues. Involving public pharmacies may introduce risks of medication errors and inaccurate documentation, as outlined in the Swiss cheese model (42). Loss of information may contribute to misjudgments regarding the actual medication regime. This limitation serves a crucial purpose in highlighting the necessity for medication.

Thirdly, self-procuring over-the-counter medication adds complexity to medication management. For example, the use of herbal agents and synthetic sleeping pills without physician consultation represents unreported cases, further complicating medication assessment.

An optimum individual performance capacity is required to be effective during military service. Understanding the diverse physiological characteristics and sex-specific pharmacology may offer further insights into the relationship between medication and performance, particularly in individual cases. Due to the limited number of female patients, such considerations could not be assessed.

## 5 Conclusion

This study underscores the importance of considering medication in the rehabilitation of middle-aged patients regarding physical performance as one major rehabilitation component. Among PTSD patients, there was a notable decline in maximum performance of LTM, which was not statistically significant in somatically impaired patients or in the overall cohort.

Polypharmacy, present in nearly half of all PTSD patients receiving medication (Figure 1b), may serve as a potential precursor to the emergence of physical performance limitations, although a direct correlation between medication and performance cannot be discerned. However, comparison to somatically afflicted individuals with polypharmacy (Figure 1c) suggested that either the functional deficit itself or the specific medication used may impact performance. This clearly indicates demand for future research and suggests a need for professional support in medication issues. This finding prompts reconsideration regarding the enhancement of therapeutic services within the established repertoire of rehabilitative measures, as pharmaceutical counseling has not yet been a firmly implemented service in either civilian or military rehabilitation (18, 45, 46).

Nevertheless, the complexity of causal relationships poses a considerable therapeutic and rehabilitative challenge for patients with mental illness (41). The results from a longitudinal observation of PTSD patients showed neither a positive effect of rehabilitation measurements nor a decline in physical performance (47).

Depressive disorders caused by operational injuries not only entail the risk of soldiers being restricted but also worsen their quality of life to a similar extent as severe chronic physical illnesses (34). The prognosis and course of physical impairment can be negatively influenced by a depressive disorder (34). The treatment and control of depressive symptoms are multifactorial, and medication might be an important pillar of patient rehabilitation (34). Drug therapy must be carefully planned, and the advantages and disadvantages must be weighed against each other (48, 49).

## Data Availability

The data is not readily available and can be requested upon reasonable requests. Further queries can be directed to the corresponding author.
